# *Streptococcus mitis* Induces Conversion of *Helicobacter pylori* to Coccoid Cells during Co-Culture In Vitro

**DOI:** 10.1371/journal.pone.0112214

**Published:** 2014-11-11

**Authors:** Yalda Khosravi, Yakhya Dieye, Mun Fai Loke, Khean Lee Goh, Jamuna Vadivelu

**Affiliations:** 1 Department of Medical Microbiology, Faculty of Medicine, University of Malaya, Kuala Lumpur, Malaysia; 2 Vice-chancellor's Office, University of Malaya, Kuala Lumpur, Malaysia; 3 Department of Medicine, Faculty of Medicine, University of Malaya, Kuala Lumpur, Malaysia; Aarhus University, Denmark

## Abstract

*Helicobacter pylori* (*H. pylori*) is a major gastric pathogen that has been associated with humans for more than 60,000 years. *H. pylori* causes different gastric diseases including dyspepsia, ulcers and gastric cancers. Disease development depends on several factors including the infecting *H. pylori* strain, environmental and host factors. Another factor that might influence *H. pylori* colonization and diseases is the gastric microbiota that was overlooked for long because of the belief that human stomach was a hostile environment that cannot support microbial life. Once established, *H. pylori* mainly resides in the gastric mucosa and interacts with the resident bacteria. How these interactions impact on *H. pylori*-caused diseases has been poorly studied in human. In this study, we analyzed the interactions between *H. pylori* and two bacteria, *Streptocccus mitis* and *Lactobacillus fermentum* that are present in the stomach of both healthy and gastric disease human patients. We have found that *S. mitis* produced and released one or more diffusible factors that induce growth inhibition and coccoid conversion of *H. pylori* cells. In contrast, both *H. pylori* and *L. fermentum* secreted factors that promote survival of *S. mitis* during the stationary phase of growth. Using a metabolomics approach, we identified compounds that might be responsible for the conversion of *H. pylori* from spiral to coccoid cells. This study provide evidences that gastric bacteria influences *H. pylori* physiology and therefore possibly the diseases this bacterium causes.

## Introduction

*Helicobacter pylori* (*H. pylori*) is a major gastric pathogen that has been associated with humans for more than 60,000 years [1]. Most of *H. pylori*-infected individuals develop an asymptomatic gastritis and can harbor this bacterium for their lifetime. In a subset of hosts, *H. pylori* causes different gastric diseases including dyspepsia, ulcers and gastric cancers. Disease development depends on several factors including the infecting *H. pylori* strain, environmental and host factors [2], [3]. Another factor that is emerging as playing an important role in *H. pylori*-caused diseases is the host gastric microbiota. The role of gastric microbiota was previously overlooked because of the belief for long that human stomach was a hostile environment that cannot support microbial life. Studies subsequent to *H. pylori* discovery in 1981 showed that in fact human stomach constitutes a very diverse and complex ecosystem with a bacterial density comparable to that of the duodenum [4]. Gut microbiota plays important roles in several host functions including energy harvest and storage from the diet [5], development and regulation of the gut-associated mucosal immune system [6], regulation of the central nervous system [7], detoxification of xenobiotics and carcinogens, and protection against colonization by pathogens [8]. Although the gastric microbiota has been less studied than the microbiota in other gut sites, it is obvious that its composition and diversity are crucial for gut homeostasis.

Once established, *H. pylori* mainly resides in the gastric mucosa, a site that has a specific microbiota closely associated with the host [9]. *H. pylori* continuously interacts with the resident gastric bacteria, which affect not only *H. pylori* colonization but also the immune response to the infection [10]. Although not formerly demonstrated, it is likely that these interactions influence *H. pylori* colonization and disease development. This question has been poorly addressed in humans. However, studies in animal models of *H. pylori* infection have provided important insights. Studies in gerbil identified gastric bacteria that inhibited *H. pylori* colonization while others were enriched in *H. pylori*-infected animals [11]–[13]. Additionally, long-term infection by *H. pylori* significantly modified the composition of the gastric microbiota of gerbils [14]. A similar observation was made in mice where *H. pylori* infection altered the composition and diversity of the gastric flora [15]. In a mouse model of gastric cancer, *H. pylori*-induced carcinogenesis was delayed in the absence of the microbiota suggesting a role for gastric bacteria in the development of the disease [16], [17]. Studies that analyzed the gastric microbiota of humans infected with *H. pylori* present the limitation of including a small number of patients and have yielded conflicted results [18]. However it can be hypothesized that the modification induced by *H. pylori* colonization including elevation of gastric pH, destruction of epithelial cells and production of metabolites favor the development of certain bacterial species and inhibit others.

In this study, we wanted to investigate the interactions between *H. pylori* and two bacteria, *Streptoccus mitis* and *Lactobacillus fermentum*, which have been isolated from gastric biopsies of both healthy and gastric disease human patients including *H. pylori*-infected individuals [4], [9], [19], [20]. *S. mitis* is a member of the human oral microbiota [21]. Considered for long as a commensal, *S. mitis* is presently viewed at least as an opportunistic pathogen as evidenced by several studies that have demonstrated its involvement in oral and systemic diseases [22]. Interestingly, *S. mitis* was found to be significantly enriched in the stomach of atrophic gastritis and gastric cancer patients [19]. *L. fermentum* belongs to the group of GRAS (Generally Regarded As Safe) lactic acid bacteria. It is a member of the human gastrointestinal microbiota and strains of *L. fermentum* have shown probiotic properties providing protection against respiratory infections [23]–[25] or being used in functional food [26]. Probiotics have recently attracted interest for the treatment of *H. pylori* infection, several lactic acid bacteria showing anti-*H. pylori* properties and can possibly provide an alternative to address the increase of antibiotic resistance [27]. We have found that *S. mitis* produced and released factors that induce coccoid conversion of *H. pylori* cells during co-culture *in vitro*. In contrast, both *H. pylori* and *L. fermentum* released products that improved *S. mitis* survival during the stationary phase of growth. These interactions possibly impact on the diseases caused by *H. pylori* and could explain the increase of *S. mitis* cells in the stomach of certain gastric disease patients.

## Materials and Methods

### Bacterial strains, growth conditions and co-culture assay

**Figure 1:**
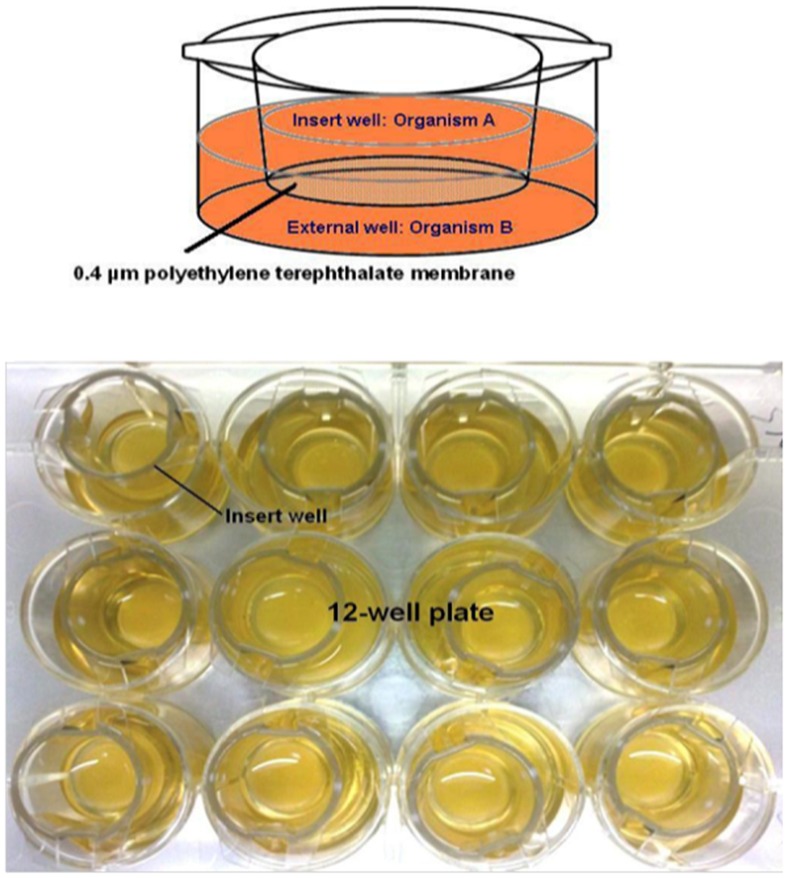
*In vitro* co-culturing system to study the interactions between *H. pylori*, *S. mitis* and *L. fermentum*. One bacterium was inoculated in a well of a 12-well plate while the other bacterium was contained in a cell culture insert placed inside the well. The two compartments were separated by a 0.4 µm membrane that prevented physical contacts between the bacteria while allowing the diffusion of macromolecules.

*H. pylori* strain NCTC 11637, *S. mitis* strain ATCC 6249 and *L. fermentum* strain ATCC 8289 were obtained from the American Type Culture Collection (ATCC, USA). *H. pylori* strain UM032 is a clinical isolate from the University of Malaya Medical Centre, Kuala Lumpur, Malaysia that was previously described [28]. All the bacteria were grown on chocolate-agar plate or in Brain Heart Infusion (BHI) broth supplemented with 0.4% yeast extract and 1% β-cyclodextrin, and incubated at 37°C in a humidified incubator with 10% CO_2_. This microaerophilic condition is needed for growth of *H. pylori in vitro* but is not a requirement for *S. mitis* and *L. fermentum*. However, for consistency, we grew all the organisms in the same conditions throughout the study. For bacterial co-culture, we used cell culture inserts (BD Biosciences, San Jose, CA, USA) that can be placed inside the wells of a12-well plate (BD Biosciences, San Jose, CA, USA) (Fig. 1). One bacterial culture was inoculated in the 12-well plate while the other microorganism was placed inside the insert. The two co-culture compartments were separated by a polyethylene terephathalate (PTE) membrane with 0.4 µm pores that prevented physical contact between the two bacteria while allowing free diffusion of macromolecules. To verify the absence of bacterial crossing of the membrane between the 2 compartments, BHI cultures of *H. pylori*, *S. mitis* and *L. fermentum* were inoculated in inserts that were placed on wells of a 12-well plate containing fresh BHI broth and incubated for 5 days in an incubator as described above. Presence of bacterial cells in the wells was verified daily by plating 100 µl of the broth onto chocolate-agar plates that were incubated at 37°C in an incubator with 10% CO_2_. No bacterial growth was detected in these tests for the 5 consecutive days (not shown). For co-culture assay with *H. pylori*, cells from a 2–3 day old chocolate-agar plate were used to make a suspension of OD_600_ ∼0.02 (10^6^–10^7^ cfu/ml). Two ml of suspension of strain NCTC 11637 or of strain UM032 were distributed in each well of a 12-well plate. A cell culture insert containing 0.5 ml of a suspension of *S. mitis* or of *L. fermentum* at OD_600_ ∼0.008 (10^5^–10^6^ cfu/ml) prepared from an overnight culture in BHI broth was then placed in each well. We found these proportions between the slow growing *H. pylori* and the faster growing *S. mitis* and *L. fermentum* to give the most reproducible results. For co-culture of *S. mitis* and *L. fermentum*, each compartment received a bacterial suspension at OD_600_ ∼0.008. The co-cultured bacteria were incubated at 37°C in a humidified incubator with 10% CO_2_ for 7 consecutive days. At each day, dilutions from each of the co-culture compartments were plated onto chocolate-agar plates that were incubated at 37°C as described above. The bacterial count was determined after 1 day for *S. mitis* and *L. fermentum*, and after 3 days for *H. pylori*. Additionally, *H. pylori* cells were recovered during both mono and co-culture, Gram-stained and examined at the microscope to inspect the morphology of the bacteria. Each co-culture experiment was repeated at least three times.

### Effect of supernatant on bacterial culture

Supernatant of *H. pylori* cultures were obtained by inoculating BHI broth with cells from a 2–3 day old chocolate-agar plate and incubated at 37°C as described above. After 1, 2 and 4 days of growth, the culture was centrifuged at 5,000 *g* for 5 min. and the supernatant recovered and filtered with a 0.22 µm syringe filter (Terumo Europe N.V., Leuven, Belgium). The filtered supernatant was used immediately. *S. mitis* supernatant was prepared similarly except that the BHI broth was inoculated (1/100 dilution) from an overnight liquid culture. To test the effect of the supernatants, 2 ml of *H. pylori* and of *S. mitis* suspension in BHI broth at OD_600_ ∼0.02 and ∼0.008 respectively, prepared as described above were distributed in each well of a 12-well plate. Five hundred microliters of filtered supernatant of 1-, 2- and 4-day old cultures of the other bacterium were added to the wells and the plates incubated at 37°C as described above. At each day, dilutions of the cultures were plated onto chocolate-agar plate and incubated at 37°C and the bacterial count determined as described above. Additionally, *H. pylori* cells were recovered, Gram-stained and examined at the microscope to monitor the presence of coccoids.

### Determination of bacterial viability

Bacterial viability was assessed by determining the metabolic activities of bacterial cells. For this purpose, the levels of cellular ATP were measured using the luciferase-based BacTiter-Glo assay kit (Promega, Madison, WI, USA) according to the manufacturer's recommendations. Briefly, 100 µl of bacterial cells from monocultures and from each compartment of co-cultures were separately mixed with 100 µl of BacTiter-Glo Reagent, incubated for five minutes at room temperature and the luminescence measured using a microplate reader (Varioskan Flash, Thermo Scientific, USA). The results were expressed as relative light units. To determine the baseline ATP content of the bacteria (level of ATP in dead cells), cells from a bacterial culture at OD_600_ ∼1 were pelleted, then re-suspended in an equal volume of 10% formalin. The ATP levels in these formalin-killed bacteria were measured immediately after mixing and at different time points up to 4 hours.

### Metabolite extraction

Metabolites were extracted from culture supernatants using methanol. Three hundred microliters of cold methanol was added to 100 µl of supernatant in a 1.5 ml Eppendorf tube. The sample was vortexed for 1 minute, incubated on ice for 30 minutes and then centrifuged at 8000 g, 4°C for 10 minutes. The supernatant was transferred to a new tube and vacuum-evaporated using a Labconco Refrigerated Centrivap concentrator (Kansas City, MO, USA) at 4°C. The sample was dissolved in mobile phase (95∶5 acetonitrile:water) immediately before analysis.

### Metabolite profiling by mass spectrometry

Liquid chromatography-mass spectrometry (LC-MS) analysis was performed on a 1260 Infinity Quaternary LC System coupled with a 6540 Quadrupole Time-of-Flight (Q-TOF) mass spectrometer with Dual Agilent Jet Stream Electrospray Ionization (Dual AJS ESI) ionization source (Agilent Technologies, Santa Clara, CA, USA). Samples were separated using the ZORBAX Eclipse Plus C18 Rapid Resolution High Throughput (RRHT) 2.1×100 mm 1.8 µm column (Agilent Technologies) and analyzed in both positive and negative ionization modes. For positive mode experiments, the mobile phases were water with 0.1% formic acid (A) and acetonitrile with 0.1% formic acid (B). A linear gradient was run from 2% to 98% B over 25 minutes, at 0.5 µl/min. In negative mode, the mobile phases were water with 1 mM ammonium fluoride (A) and acetonitrile (B). Similarly, a linear gradient was turn from 2% to 98% B over 25 minutes, with a flow rate of 0.5 µl/min. The injection volume was 3 µL and three injections were made for each sample. ESI conditions were spray voltage 3.0 kV, gas temperature, 300°C, drying gas, 8 L/min, nebulizer 35 psig, VCap 3500 V, fragmentor 175 V and skimmer 65 V. The instrument was set to acquire over the m/z range of 100–1700 with an acquisition rate of 1 spectra/s. Three biological replicates were analyzed for each sample.

### Data processing and statistical analysis

Spectrum was extracted using the MassHunter Qualitative Analysis software (version B.06.00) (Agilent Technologies). Statistical analysis was performed by Mass Profiler Professional (MPP) software (version 3.12.61) (Agilent Technologies). For normalization, entities were baselined to the median of all samples. This entity list was used for statistical analysis by applying unpaired T-test (one-way ANOVA, asymptotic p-value<0.05) and Benjamini-Hochberg FDR of 1.0% as multiple testing corrections. Entities were compared between samples by fold change in relative intensity.

## Results

To study the interactions between *H. pylori*, *S. mitis* and *L. fermentum* during growth *in vitro*, we established a co-culture method in which 2 bacterial species were grown in 2 compartments separated by a membrane that allowed exchange of diffusible molecules produced and released by the bacteria while preventing them from making a physical contact. In these assays, we wanted the cell densities in the 2 compartments to be as close as possible. For this we had to overcome 2 constraints: (i) *H. pylori* slowly grows *in vitro* while *S. mitis* and *L. fermentum* are fast growing organisms and (ii) in our growth conditions, inocula of high density were needed for *H. pylori* cultures to expand. We performed preliminary tests and from the results we chose starting inocula of *H. pylori* at 10^6^–10^7^ cfu/ml and of *S. mitis* and *L. fermentum* at 10^5^–10^6^ cfu/ml and monitored the cultures from 1 to 7 days after inoculation.

### Streptococcus mitis induces growth arrest of Helicobacter pylori cultures

**Figure 2:**
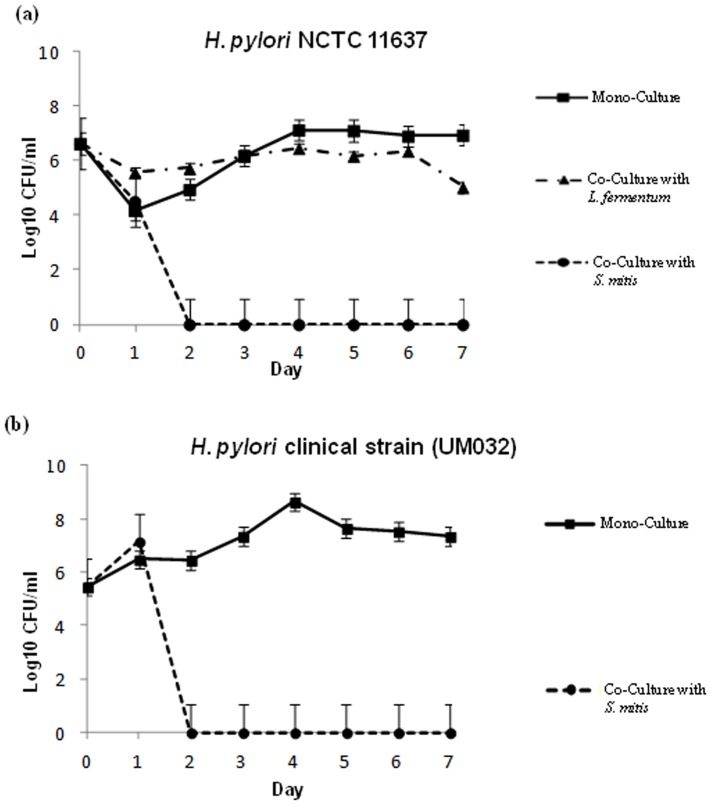
Effect of *S. mitis* and of *L. fermentum* on *H. pylori* growth during co-culture. *H. pylori* reference (NCTC 11637, (a)) and clinical (UM032, (b)) strains were grown alone or co-cultured with *S. mitis* or with *L. fermentum*. At the times indicated, colony forming unit of *H. pylori* were calculated by plating dilutions of the cells onto chocolate-agar plates. Each point shows the means and standard deviation of triplicated experiments.

*H. pylori* mono-cultures increased in cell density by ∼2 logs between day 1 and day 4 following inoculation and then stabilized up to day 7 (Fig. 2a). Interestingly, when co-cultured with *S. mitis*, cell density of *H. pylori* cultures dramatically dropped from day 1 and viable cells could not be detected after two days of co-culture (Fig. 2a). This phenomenon was not strain specific since both *H. pylori* UM032 [28], a clinical isolate and *H. pylori* NCTC, a laboratory strain displayed the same behaviour (Fig. 2b). In contrast to *S. mitis*, co-culture with *L. fermentum* did not affect the growth of *H. pylori* cells that was comparable to that of a mono-culture (Fig. 2a). These results suggest that *S. mitis* specifically inhibits growth of *H. pylori* cells in co-culture.

### Presence of *Helicobacter pylori* or of *Lactobacillus fermentum* improves the survival of *Streptococcus mitis* in the stationary phase

**Figure 3:**
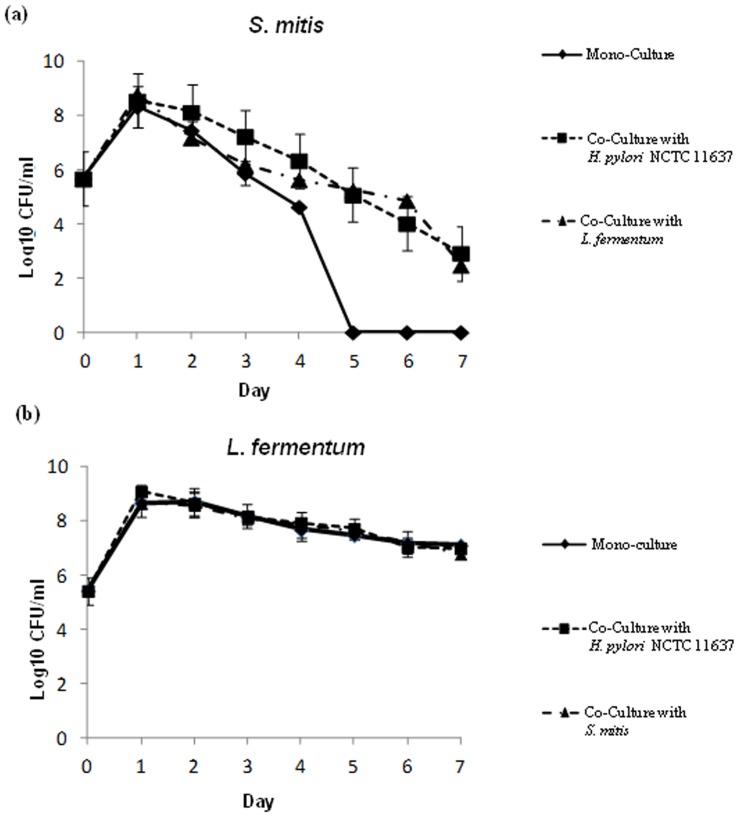
Growth of *S. mitis* and of *L. fermentum* during mono- and co-culture. *S. mitis* (a) and *L. fermentum* (b) were grown alone or co-cultured with the indicated bacteria. At the times indicated, colony forming units were calculated by plating dilutions of the cells onto chocolate-agar plates. Each point shows the mean and standard deviation of triplicated experiments.

We next analysed the effect of *H. pylori* on *S. mitis* during co-culture of the two bacteria. One day after inoculation, *S. mitis* mono-cultures already reached the stationary phase and the cell density dropped by ∼1 log per day until day 4 (Fig. 3a). From day 5, culturable cells could not be obtained anymore (Fig. 3a). In contrast, during co-culture with *H. pylori*, *S. mitis* cells were detectable until the end of the experiment at day 7, and although the cell densities continued to drop, the decrease was <1 log and viable cells could be cultured on days 5–7 when culturable cells could not be isolated from the mono-culture (Fig. 3a). Interestingly, *L. fermentum* displayed the same effect as *H. pylori* when co-cultured with *S. mitis* (Fig. 3a). These results suggest that both *H. pylori* and *L. fermentum* release products that promotes cultivability of *S. mitis* cells during the stationary phase of growth *in vitro*. To complete the analysis of the effects of the three bacteria on each other during co-culture, we monitored the growth of *L. fermentum* in the presence of *H. pylori* and of *S. mitis*. *L. fermentum* co-cultured with either species displayed a growth pattern similar to that of mono-cultures of the bacterium (Fig. 3b). All together these results suggest that *H. pylori* and *L. fermentum* released diffusible factors that promoted survival of *S. mitis* during the stationary phase of *in vitro* culture.

### Streptococcus mitis induces conversion of Helicobacter pylori to coccoid

**Figure 4:**
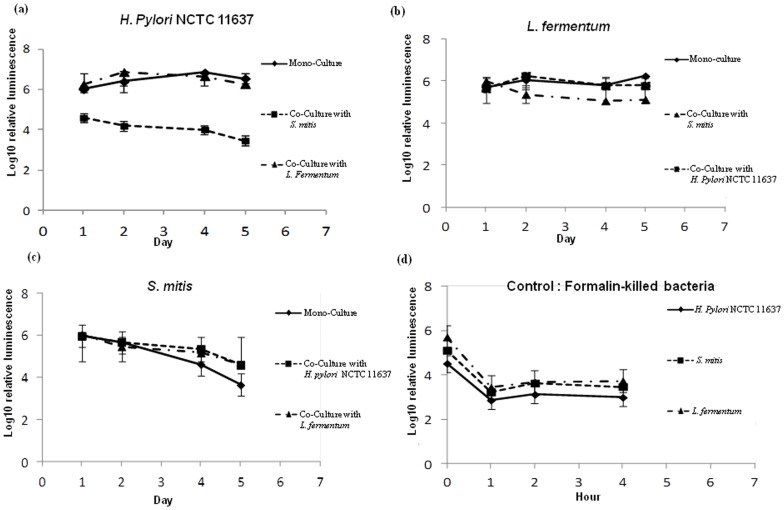
Determination of bacterial cell viability during mono and co-culture. The ATP levels were measured in *H. pylori* NCTC 11637 (a), *L. fermentum* (b) and *S.mitis* (c) cells during mono- and co-culture after 1, 2, 4 and 5 days of incubation. (d), ATP level in formalin-killed bacteria 0, 1, 2, and 4 hours after treatment.

**Figure 5:**
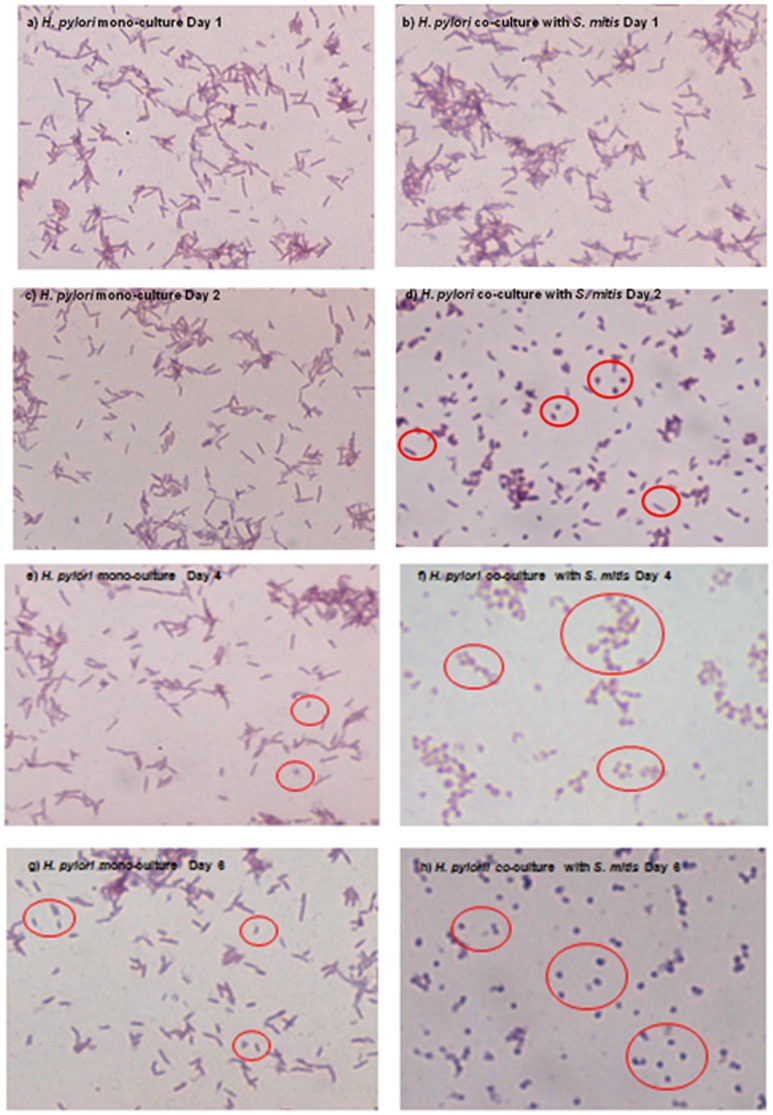
Morphology of *H. pylori* cells in mono and co-culture. *H. pylori* NCTC 11637 cells in monoculture ((a), (c), (e) and (g)) and in co-culture with *S. mitis* ((b), (d), (f) and (h)) were recovered after 1 ((a) and (b)), 2 ((c) and (d)), 4 (e) and (f)) and 6 ((g) and (h)) of growth. The bacteria were Gram-stained and microscopically examined with a 1000X magnification.

We wanted to further investigate the growth arrest of *H. pylori* cells co-cultured with *S. mitis*. We first verified whether the *H. pylori* cells were still alive or were killed by measuring their level of ATP, an indicator of metabolic activity. *H. pylori* cells in mono-cultures or co-cultured with *L. fermentum* had similar levels of ATP (Fig. 4a). When co-cultured with *S.mitis, H. pylori* cells displayed levels of ATP significantly lower than in mono-cultures at days 1, 2, 4 and 5 the times points tested in this experiment (Fig. 4a). However, these levels of ATP were significantly higher than the ones found in formalin-killed bacteria (Fig. 4d) suggesting that *H. pylori* cells in co-culture with *S. mitis* were alive. As controls, we measured the ATP contents of *S. mitis* (Fig. 4c) and *L. fermentum* (Fig. 4b) that, contrary to *H. pylori*, did not experience growth arrest. In both mono- and co-culture conditions, the bacteria displayed similar levels of ATP that were comparable to that in *H. pylori* cells in monoculture (Fig. 4a) but higher that ATP levels in *H. pylori* co-cultured with *S. mitis*. Collectively, these results showed that *H. pylori* cells that experienced growth arrest when co-cultured with *S. mitis* were alive though with a reduced metabolic activity. *H. pylori* is known to convert from a spiral to a coccoid shape in adverse conditions such as nutrient limitation, environmental stress, or presence of antibacterial compounds [29]. Since coccoid *H. pylori* cells are alive but non-culturable, we wanted to verify whether the presence of *S. mitis* induced this morphological change. For this, we performed microscopic examination of Gram-stained *H. pylori* cells grown alone or co-cultured with *S. mitis*. *H. pylori* cells in monoculture appeared in bacillary form at days 1, 2 and 4 (Figs. 5a, 5c and 5e), while coccoid cells were detected at Day 6 (Fig. 5g) likely indicating nutrient limitation in the medium. In contrast, during co-culture with *S. mitis*, coccoid *H. pylori* cells were predominant at Day 2 (Fig. 5d), a time point that corresponded to failure to obtain culturable bacteria (Fig. 2a), and were exclusively present at days 4 and 6 (Figs. 5f and 5h). These results clearly showed that *S. mitis* induced morphological conversion of *H. pylori* to coccoid cells.

### Coccoid conversion of *Helicobacter pylori* and extended survival of *Streptococcus mitis* during co-culture are mediated by diffusible factors released by the two bacteria

**Figure 6:**
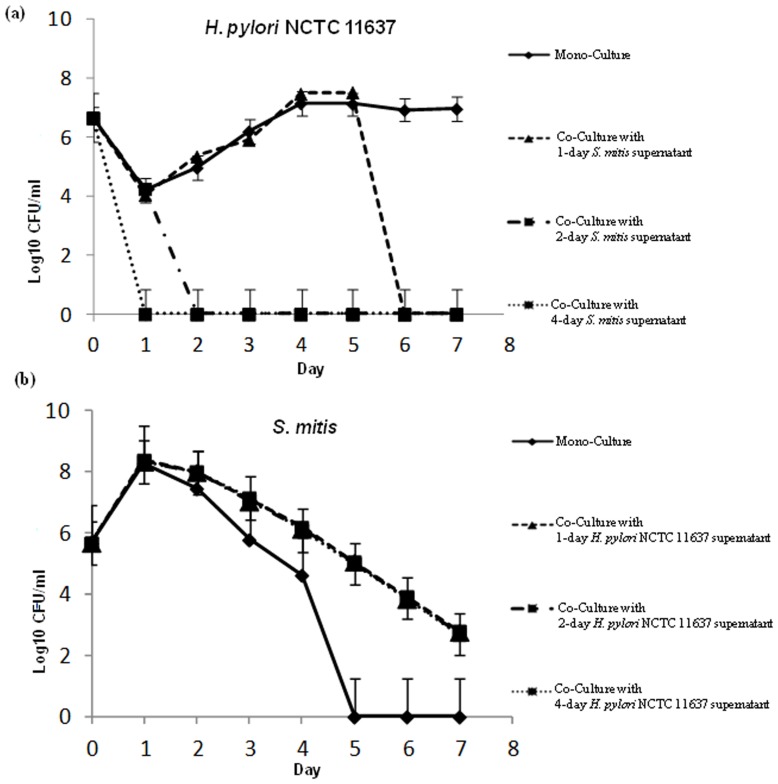
Effect of supernatant from the other bacterium on *H. pylori* and *S. mitis*. Mono-cultures of *H. pylori* (a) and of *S. mitis* (b) were supplemented with 1-, 2- or 4-day old supernatants from the other bacterium or left untreated. The colony forming units were determined by plating at the indicated time points.

The effects exerted by *H. pylori* and *S. mitis* on each other during co-culture might result from diffusible factors or metabolites the two bacteria produced. Alternatively, these effects could be explained by the difference in growth speed between the two organisms. *S. mitis* growing faster, it may deplete the medium in *H. pylori* compartment thus acquiring more nutriment while applying a stress on *H. pylori* that converts to coccoid. To differentiate between these possibilities, we compared the growth of each bacterium in the absence or in the presence of supernatants from 1-, 2- or 4-day old culture of the other organism. *H. pylori* cells supplemented with a 1-day *S. mitis* supernatant grew similarly as non-supplemented bacteria until day 5, then culturable cells could not be obtained at later time points (Fig. 6a). In contrast, in the presence of a supernatant from a 2-day old *S. mitis* culture, *H. pylori* cells experienced a growth arrest after two days of culture (Fig. 6a). The growth arrest was even more dramatic in the presence of a 4-day *S. mitis* supernatant that resulted in a failure to obtain culturable *H. pylori* cells at day 1 (Fig. 6a). These results indicated that growth arrest and coccoid conversion in *H. pylori* was mediated by a factor(s) released or a metabolite(s) produced by *S. mitis* that accumulate(s) during the stationary phase. Similarly to co-culture, survival of *S. mitis* cells was improved by addition of supernatants from *H. pylori* cultures (Fig. 6b). We did not observe differences in *S. mitis* survival depending on the age of the *H. pylori* culture. These results indicated that the benefit conferred to *S. mitis* is mediated by a diffusible product(s) present in *H. pylori* supernatant before the stationary phase of growth.

### Identification of small moleculs involed in interaction of *H. pylori* and *S. mitis* by LCMS

To identify the factors that mediate coccoid conversion of *H. pylori* and survival of *S. mitis* during late exponential growth phase, we determined the metabolite profiles of supernatants from 1-, 2- and 4-day old monocultures and co-cultures of the two bacteria by LC/MS (see materials and methods). Metabolites that were statistically significantly up-regulated by more than two folds at any time point in triplicated experiments could be classified into three groups (Table 1). Group 1 comprises metabolites that were detected in the co-culture and in either of the mono-cultures (Table 1). Group 2 was constituted by compounds found only in the co-culture while Group 3 contained products from *S. mitis* and/or *H. pylori* mono-culture that were absent in the co-culture (Table 1). Metabolites involved in *H. pylori* conversion to coccoid or the extended survival of *S. mitis* are likely to belong to Group 1 while compounds in groups 2 and 3 mainly demonstrate interactions between *H. pylori* and *S. mitis* during co-culture. We reasoned that a compound that induced *H. pylori* conversion to coccoid should be present in supernatant from *S. mitis* and possibly from co-culture. Additionally, this compounds should start accumulating at day 2 since supernatant from this time point and not from a 1-day old culture induced coccoid conversion (Fig. 6a). Only one compound, a Tenovin-6-like molecule, that belongs to group 1 fulfilled these requirements (Table 1). The Tenovin-6-like compound was significantly up-regulated in *S. mitis* supernatant at day 2 (8019 fold change) and drastically increased at day 4 (21234 fold change). This molecule was also significantly up-regulated in supernatants from co-culture at the three time points tested (Table 1).Three other metabolites could theoretically be involved in *H. pylori* conversion to coccoid. The first of these metabolites (KDNα2-3Galβ1-3(KDNα2-6)GalNAcβ1-4Galβ1-4Glcβ-Cer) was similar to a compound that belongs to the class of gangliosides and, similarly to the Tenovin-6-like molecule, was significantly up-regulated in *S. mitis* supernatant at day 2 and increased at day 4 (Table 1). However, this metabolite was absent in supernatants from the co-culture. The two other compounds were similar to Sulfoglycolithocholate, a secondary bile salts metabolite and to Caracurine V, a plant derived antimicrobial, respectively. These two compounds were detected in the co-culture but were absent in the mono-cultures (Table 1). To identify metabolites involved in *S. mitis* survival at the exponential growth phase, we reasoned that such compounds were to be expected in supernatants from *H. pylori* cultures at the three time points since these supernatants equally conferred the extended survival phenotype to *S. mitis* (Fig. 6b). None of the metabolites were up-regulated at least 2 folds in the three time points in *H. pylori* supernatant. A total of six compounds were moderately up-regulated in *H. pylori* supernatants (1.8 to 5.6 fold change). An additional metabolite similar to 25-O-Deacetyl rifabutin N-oxide displayed a 77 fold change but only at day 4 (Table 1). Although these compounds could theoretically play a role in *S. mitis* survival we believe their involvement in this phenomenon to be unlikely.

**Table 1:** List of metabolites significantly up-regulated in supernatants of *H. pylori* NCTC 11637 and *S. mitis* during mono- and co-culture.

Metabolite	Fold Change								
	*H. pylori*			*S. mitis*			Co-culture		
	Day 1	Day 2	Day 4	Day 1	Day 2	Day 4	Day 1	Day 2	Day 4
**Group 1**									
LysoPE (24:6(6Z,9Z,12Z,15Z,18Z,21Z)/0:0)				3.26	3.20	4.82	3.80	3.35	3.28
Tenovin-6					8019	21234	18094	7933	7944
									
Ganglioside GM1 (d18:0/14:0)	1.79	2.07	1.83				1.78	2.05	1.82
Lithocholate 3-O-glucuronide		2.23	5.59				2.19	4.57	7.08
**Group 2**									
2,2-Dimethyl-3,4-bis(4-methoxyphenyl)-2H-1-benzopyran-7-ol acetate							1.93	4.88	6.74
LysoPE(0:0/14:0)							2.02	2.44	2.47
2-O-hexadecanoyl-3-O-(2,4S,6S-trimethyl-2E-pentacosenoyl)-6-O-(2S,4S,6S-trimethyl-3S-hydroxy-tetracosanoyl)-2′-O-(2,4S,6S-trimethyl-2E-tetracosenoyl)-4′-O-(2,4S,6S-trimethyl-2E-docosenoyl)-α,α-trehalose								2.01	2.05
PG(12:0/16:0) +12.243238							2.09	2.46	2.47
PG(12:0/16:0) +12.243523							2.09	2.48	2.50
Sulfoglycolithocholate - 0.76514286								6.78	87062
Sulfoglycolithocholate								513	0.76
Caracurine V - 0.5971875							5725	4536	14336
Caracurine V							33608	4546	3088
PI(12:0/18:3(9Z,12Z,15Z))							4.75	46490	51388
PI(12:0/18:3(9Z,12Z,15Z)) - 0.72683334							4.75	7289	52393
**Group 3**									
Mycalamide A				3.83	4.20	3.68			
PG(12:0/16:0) +12.247833:3				2.13	2.40	2.48			
PG(12:0/16:0) +12.247833				2.14	2.41	2.49			
KDNα2-3Galβ1-3(KDNα2-6)GalNAcβ1-4Galβ1-4Glcβ-Cer(d18:1/26:0)					26.85	25895			
KDNα2-3Galβ1-3(KDNα2-6)GalNAcβ1-4Galβ1-4Glcβ-Cer(d18:1/26:0) - 11.1105^*^					26.85	25882			
7-Hydroxychlorophyll a	1.93	2.32	2.05	2.02	2.11	2.07			
Ankorine	2.06	2.57	1.83	2.25	2.43	2.20			
Alpha-Heptasaccharide		1.74			2.18				
Manzamine A		2.07	1.63						
25-O-Deacetyl rifabutin N-oxide			77.16						

## Discussion

In this study, we analyzed the interactions, during growth *in vitro*, between *H. pylori* and two bacteria, *S. mitis* and *L. fermentum* that have been isolated from the stomach of both healthy and *H. pylori*-infected gastric disease patients [4], [9], [19], [20]. Using a co-culture method, we found that *S. mitis* produced and released one or more diffusible factors that directly or indirectly induce coccoid conversion of *H. pylori* cells. In contrast, both *H. pylori* and *L. fermentum* secreted factors that promote survival of *S. mitis* during the stationary phase of growth. We did not find any effect of *H. pylori* or *S. mitis* on the growth of *L. fermentum* during co-culture. To identify the factors responsible for coccoid conversion of *H. pylori* and for survival of *S. mitis* in the stationary phase, we performed metabolomics analysis of supernatants from mono- and co-cultures of *H. pylori* and *S. mitis*. We detected a few compounds that could possibly be involved in *H. pylori*'s morphological conversion while we did not find molecules that match the phenotype conferred to *S. mitis* by co-culture or *H. pylori* supernatant supplementation. It should be noted that both *H. pylori* conversion to coccoid and *S. mitis* survival in the stationary phase could be mediated by proteins secreted by these bacteria. Such factors could not be detected by the LC/MS approach we used in this study.

One of the compounds we detected was similar to Tenovin-6 an anticancer molecule that was first identified in a screen for p53 activators (Lain et al 2008). p53 is a tumor suppressor encoded by a gene that is the most mutated gene in cancer. Tenovin-6 is currently subjected to intensive studies because of promises this molecule held in cancer treatment [30]–[32]. The Tenovin-6-like molecule identified in our metabolic profiling was the compound that best matched the induction of coccoid conversion of *H. pylori*. It was not significantly increased in supernatant from a 1-day old *S. mitis* mono-culture but was highly induced from day 2 and drastically increased at day 4 (Table 1). Coccoid conversion of *H. pylori* during co-culture with *S. mitis* was detected from day 2. Furthermore, 2-day old but not 1-day old *S. mitis* supernatants induced *H. pylori* coccoid conversion and this phenotype was more pronounced with supernatant from a 4 day old culture (Fig. 6a). Whether the effect of the Tenovin-6-like molecule has any significance during colonization of the stomach by *H. pylori* and *S. mitis* is unknown. However, the anticancer properties of Tenovin-6 and the possible role of coccoid *H. pylori* in tumorisation deserve much attention. Chan et *al*. analyzed gastrectomy specimens from cancer and peptic ulcer patients and found that coccoid *H. pylori* cells were enriched in adenocarcinoma compared to peptic ulcer samples [33]. Consistent with this finding, another study that compared the effects of spiral and coccoid *H. pylori* cells on gastric epithelial cells reported that coccoid *H. pylori* exerted a stronger effect on proliferation and a weaker effect on apoptosis than did spiral form [34]. These observations suggest an involvement of coccoid *H. pylori* in carcinogenesis. It is tempting to hypothesize from these observations that the Tenovin-6-like molecule produced by *S. mitis* antagonizes coccoid cells during colonization of the stomach by the two bacteria. Interestingly, during co-culture, the Tenovin-6-like molecule started accumulating at day 1 while it was detected at day 2 in *S. mitis* mono-culture (Table 1). This observation suggests that spiral *H. pylori* but not coccoid cells stimulates the production of the compound by *S. mitis*. Further investigation are needed to elucidate the effect of Tenovin-6-like molecule on *H. pylori* both *in vitro* and *in vivo*.

The findings in this study reflect the numerous interactions that take place between the members of the gastric microbiota. These interactions contribute to shaping the composition of the gastric microbiota and indirectly influence the pathogenesis of bacteria like *H. pylori*. *H. pylori* is known to undergo a morphological change from spiral to coccoid form in adverse conditions [35]–[39]. Coccoid cells are more resistant to different stresses and represent a survival form of the bacterium [40], [41]. However, how spiral *H. pylori* cells convert to coccoid *in vivo* and the role of coccoid cells in *H. pylori* pathogenesis are still unclear. Our findings point to a possible mechanism in which members of the gastric microbiota secrete factors that induce coccoid conversion of spiral *H. pylori* cells. These bacteria by this means indirectly influence *H. pylori* pathogenesis and disease outcome in infected individuals.
